# Developing critical thinking through scaffolded peer feedback: an action research on heuristic design

**DOI:** 10.3389/fpsyg.2026.1711768

**Published:** 2026-01-30

**Authors:** Xin Chen

**Affiliations:** College of Education, Qufu Normal University, Rizhao, Shandong, China

**Keywords:** critical thinking, action research, epistemic network analysis, heuristic scaffolding, peer assessment

## Abstract

**Introduction:**

Although peer assessment has been shown to promote critical thinking, its effectiveness depends heavily on structured guidance. This study aimed to design and validate a heuristic scaffolding framework embedded throughout the entire peer assessment process.

**Methods:**

Adopting an action research approach, the study conducted two iterative rounds of teaching practice. Textual data generated from peer assessments in both rounds were collected and analyzed using epistemic network analysis to quantitatively examine the co-occurrence relationships and structural connections among core elements of critical thinking, thereby evaluating the intervention effect of the scaffolding on students’ critical thinking.

**Results:**

The findings indicate that the heuristic scaffolding effectively activated various elements of learners’ critical thinking and facilitated the development of an interconnected cognitive network. While the initial scaffolding prompted the application of macro-level skills, a tendency toward “emphasizing evaluation over interpretation and reasoning” was observed. The optimized scaffolding, refined after the first round of intervention, significantly strengthened the cognitive chain from “evaluation to reasoning to self-regulation” and promoted the advancement of critical thinking to higher levels.

**Discussion:**

This study provides educators with a replicable scaffolding tool and a data-driven pathway for instructional improvement to design peer assessment activities that foster higher-order thinking.

## 1 Introduction

Critical thinking, a core competency for independent thinking and problem-solving, is essential for innovation and has thus become a vital educational objective at all levels (Halpern, 1999). It not only enables students to analyze and evaluate information more effectively but also supports their future career development and social participation. Peer assessment--emphasizing autonomy, agency, collaboration, and open-mindedness-- has emerged as a way to combine formative assessment with learning support to foster critical thinking (Topping, 2017). This approach facilitates multi-directional interactions among teachers, students, and learning content, which enhances agency and stimulates cognitive and social reflection (Li et al., 2021; Van Popta et al., 2017), thus promoting critical thinking (Chang et al., 2020; Zheng et al., 2019).

However, the act of guiding students to engage in systematic and profound reflection through the medium of peer assessment continues to present a considerable challenge (Adachi et al., 2018). Many peer assessment activities still focus on superficial evaluation, lacking coherent scaffolding for higher-order cognitive behaviors such as analysis, reasoning, interpretation, and self-regulation, which limits their substantive contribution to the development of students’ critical thinking. In response, this study analyzes the intrinsic connection between the core elements of critical thinking and peer assessment activities, and designs and develops targeted scaffolding tools to guide students in enhancing their critical thinking via peer evaluation and self-reflection.

## 2 Related works

### 2.1 Teaching of critical thinking

Critical thinking refers to the process by which individuals engage in reasonable, reflective thinking to decide what to believe or do, based on objective experience (Ennis, 1993; Facione et al., 1994). It constitutes purposeful, self-regulated judgment (Facione, 2000), and is fundamentally characterized by the integrated use of cognitive skills and metacognitive strategies (Ennis and Philosophy Documentation Center, 2011). It encompasses not only cognitive skills such as analysis, inference, interpretation, and self-regulation (Facione, 2011; Akpur, 2020; Hart et al., 2021; Wang et al., 2022), but also affective dispositions such as open-mindedness and inquisitiveness (Evans, 2008; Karakuş, 2024), and is markedly context-dependent and culturally specific (Ka Yuk Chan and Luo, 2022).

Research indicates that systematic instruction and training can effectively enhance students’ critical thinking skills (Pithers and Soden, 2000; Bensley et al., 2010; Gaviria Alzate et al., 2025). Various pedagogical approaches have been designed and validated for improving critical thinking: (1) Questioning technique training effectively guides students to pose in-depth questions, thereby cultivating their critical thinking abilities (Yang et al., 2005). (2) Problem-based learning (PBL) begins with a problem, requiring students to comprehend, analyze, generate and evaluate potential solutions, and make final decisions (Alsaleh, 2020). (3) Technology-supported interactive learning creates states of “cognitive imbalance” (e.g., error configuration-troubleshooting cycles in virtual experiments), prompting students to deepen understanding through deconstruction and reconstruction of knowledge (Salleh et al., 2012). (4) Collaborative learning employs heterogeneous grouping, task-driven activities, and structured debates to externalize thinking, reflect on differences, and achieve conceptual reconstruction through group negotiation, significantly enhancing critical thinking skills (Pithers and Soden, 2000; Gaviria Alzate et al., 2025). Overall, teaching strategies for critical thinking have evolved from traditional training to technology-enhanced and socially interactive approaches.

The design principles of social interactive methods include: first, creating cognitive dissonance to stimulate learners’ cognitive drive and metacognitive awareness, prompting reflection on their decision-making or problem-solving processes (Calleja, 2014; Xu et al., 2025); second, guiding students to consciously reflect on and actively analyze their core viewpoints. The developmental pathway of such methods generally involves three steps: (1) theoretical understanding—comprehending the principles and conceptual frameworks of critical thinking (Gelder, 2005; Kuhn, 1999); (2) contextualized practice—applying these skills in authentic scenarios (Karagöl and Bekmezci, 2015; Wang and Zhang, 2025); and (3) transfer and reinforcement—applying critical thinking skills across different domains (Halpern, 1999). Thus, the effectiveness of social interactive methods (e.g., peer assessment) highly depends on the quality of interaction design, necessitating heuristic scaffolding to guide learners in synergistically engaging cognitive skills and affective dispositions.

### 2.2 Peer assessment

Peer assessment aims to shift the dominance of learning from teachers to students (Hwang et al., 2014). It involves learners with similar backgrounds evaluating and providing multidimensional feedback on their peers’ work based on specific criteria (Topping, 1998). This process inherently reflects a social constructivist view of learning—knowledge co-construction through social interaction. A complete peer assessment cycle comprises four stages: submission, providing feedback, reviewing feedback, and revising work (Kollar and Fischer, 2010; Barahona et al., 2023). Students act both as assessors, offering constructive feedback (output phase), and as assessees, receiving and processing feedback from others (input phase) (Friess and Goupee, 2020; Zhang and Hwang, 2023). This process enhances students’ learning motivation, academic achievement, observational skills, and teamwork abilities (Nurcilin Asha and Taj Aysha, 2021; Wang and Zhang, 2025).

The cyclical process of output–reception–internalization promotes cognitive, affective, and social development. Cognitively, evaluative discrepancies trigger cognitive conflict, prompting deeper elaboration (Chang and Wongwatkit, 2024; Chen et al., 2009), directly fostering core critical thinking skills such as analysis and evaluation. Affectively, participation in peer assessment enhances intrinsic motivation (Hsia and Hwang, 2021; Hwang et al., 2014; Yang and Tsai, 2010) and self-regulated learning awareness. Socially, it strengthens collaborative and communication skills (Xiao and Lucking, 2008). However, research on peer assessment faces several challenges. There is insufficient research on the guiding mechanisms for cognitive conflict and systematic reflection. Although numerous studies confirm that evaluative differences-triggered cognitive conflict is a key driver for critical thinking (Chang and Wongwatkit, 2024; Jeong, 2003; Jiang et al., 2023), there remains a lack of systematic exploration on how to design effective conflict-guided scaffolding strategies within peer assessment.

### 2.3 The present study

The development of critical thinking is a continuum progressing from lower-order to higher-order thinking (Leng and Lu, 2020). Existing research indicates that with sustained social interaction (e.g., peer assessment), students’ critical thinking can gradually advance from relatively basic to more sophisticated levels (Zhang et al., 2022). However, most studies depend on static outcome evaluations, which are insufficient for revealing the micro-level cognitive mechanisms underlying this dynamic process (Ifenthaler, 2022). There is a particular lack of systematic intervention into and investigation of the triggering, regulation, and integration mechanisms of cognitive conflict within peer assessment activities.

To address these research gaps, this study adopts an action research approach, implementing iterative cycles of “design–implementation–reflection–revision” to systematically develop and validate a set of heuristic scaffolding for peer assessment. This scaffolding closely integrates core elements of critical thinking (e.g., analysis, inference, evaluation, and self-regulation), aiming to provide a structured, operable sociocognitive script for the peer assessment process. It guides students to continuously experience cognitive conflict, perspective integration, and metacognitive regulation through the interaction of “evaluating others” and “reflecting on oneself,” thereby promoting the advancement of critical thinking to higher levels.

Beyond examining the macro-level impact of scaffolding on critical thinking performance, this study emphasizes the collection of fine-grained data (e.g., peer assessment reports generated under scaffolding guidance). Utilizing quantitative ethnographic methods such as epistemic network analysis (ENA), it seeks to uncover the associative patterns and developmental trajectories of students’ critical thinking elements under the intervention of heuristic scaffolding at a micro level, thereby elucidating the internal mechanisms through which scaffolding facilitates the development of critical thinking.

## 3 Materials and methods

### 3.1 The intervention: the scaffolding framework

**Figure 1:**
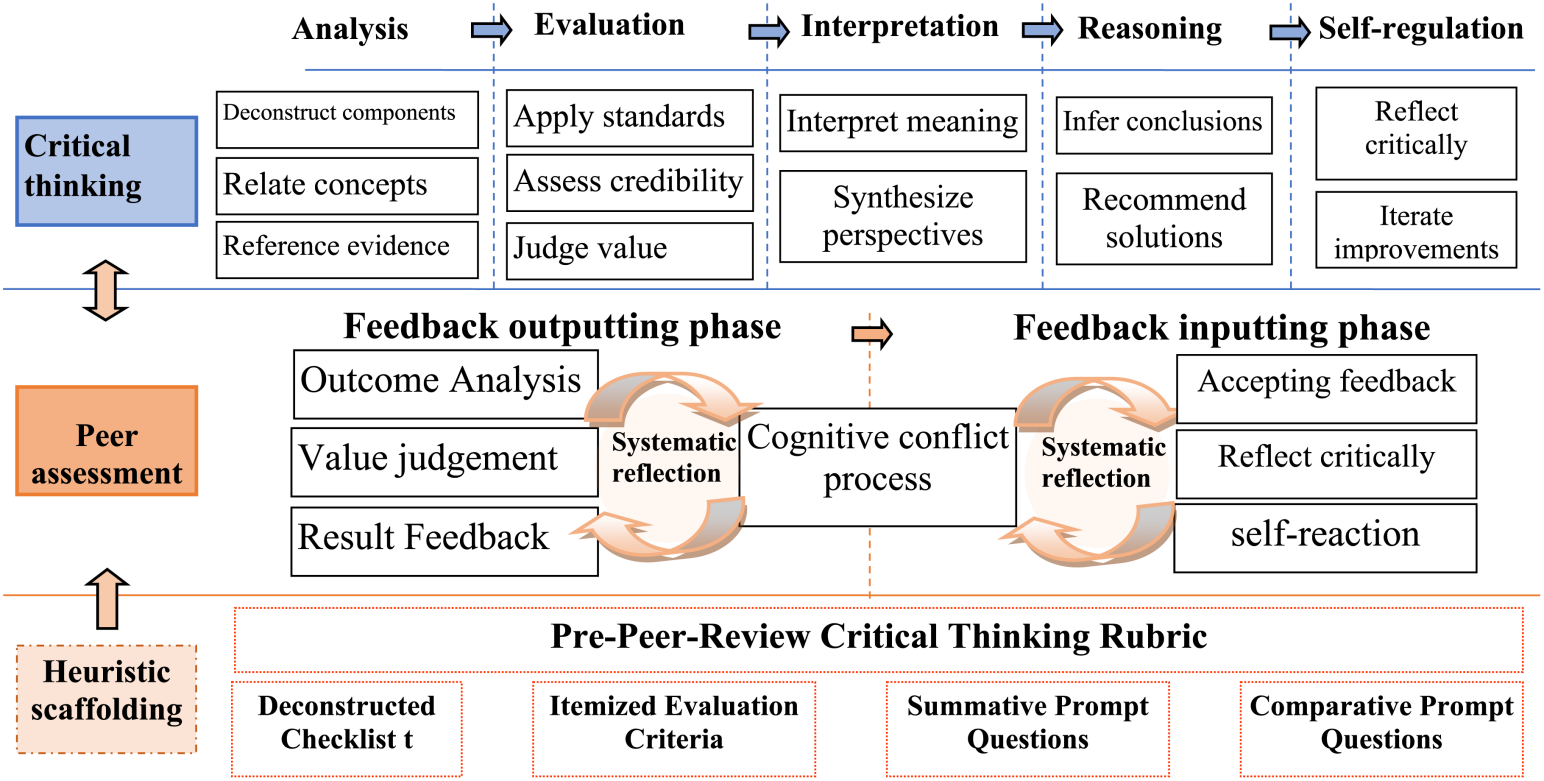
The heuristic scaffolding framework.

Critical thinking is a systematic cognitive activity that requires individuals not only to possess the ability to analyze, reason, and evaluate, but also to maintain an open, reflective, and iterative mindset. Its fundamental goal is to achieve a deep understanding of issues and make reasonable, valuable judgments and decisions through rigorous cognitive operations. Therefore, based on Facione’s (2011) critical thinking framework, this study identifies analysis, evaluation, inference, explanation, and self-regulation as the core components of critical thinking (see Figure 1).

This study conceptualizes peer assessment as a cyclical process consisting of two integrated phases: “feedback output” and “feedback input.” In the output phase, learners act as assessors, analyzing and evaluating their peers’ work to generate constructive feedback. In the input phase, learners serve as recipients, critically examining feedback received, engaging in self-regulation, and revising their own work. The core objective is to internalize external input to optimize cognitive structures. These two phases are not isolated but are connected through “cognitive conflict” and “systematic reflection,” which constitute the key mechanism through which peer assessment promotes critical thinking.

Cognitive conflict may arise when learners, as assessors, compare others’ work with their own, or as assessees, when they receive feedback that diverges from their own views, methods, or conclusions. Such conflict activates intrinsic cognitive motivation, prompting individuals to re-examine their decision-making paths and thought processes. Systematic reflection requires learners to engage in critical analysis of concepts, facts, and evidence to clarify underlying intentions without uncritically accepting authority (Dewey, 2022). In the context of peer assessment, this involves analyzing, judging, and filtering feedback from others, thereby refining one’s own cognitive framework and resolving cognitive conflicts.

To support this process, this study designed a heuristic scaffolding system centered on the core elements of critical thinking, covering the entire peer assessment process. The scaffolding is characterized by clear objectives, operable structure, and progressive guidance, and consists of the following five components:

(1) Pre-assessment Critical Thinking Qualitative Rubric: these rubric guides students to clarify the specific criteria for each dimension of critical thinking—analysis, evaluation, explanation, inference, and self-regulation—before conducting peer assessment. It activates their cognitive framework, minimizes rater bias, and provides a benchmark for subsequent feedback.
(2) Deconstructed Checklist for “Analysis”: given that peer-assessed works often possess considerable complexity, this checklist prompts learners to deconstruct the evaluation task in depth, moving beyond superficial reading. It guides them to systematically examine conceptual connections, identify evaluative criteria, and locate supporting evidence, thereby fostering systematic analysis habits and enhancing the precision and depth of feedback. It also reduces cognitive load to some extent.
(3) Itemized Evaluation Criteria for “Evaluation”: clear evaluation standards are established for each assessment item to help students make reliable judgments based on scoring criteria and articulate their reasoning. Complementary heuristic questions are designed to assist learners in assessing the validity of information, the soundness of arguments, and logical consistency, thereby triggering cognitive conflict and facilitating self-improvement.
(4) Summative Prompt Questions for “Explanation” and “Inference”: Synthesis prompts encourage evaluators to move beyond surface-level content and engage in interpretive and creative thinking—e.g., “What do you believe is the author’s core intention or underlying message?” They help connect prior knowledge with new perspectives, providing macro-level insights and constructive suggestions for assessees—e.g., “How would you improve or expand this work based on its shortcomings?” These prompts also gradually cultivate synthetic and transferable skills—e.g., “What makes this work unique compared to other related research or methods?”
(5) Comparative Prompt Questions for “Self-reflection”: Comparative prompts are designed to guide learners in systematically comparing others’ work with their own, identifying gaps in depth, breadth, or creativity, diagnosing their own deficiencies, and proactively deriving strategies for improvement. This shifts their role from passive recipients of feedback to active comparative learners, strengthening self-regulatory and iterative optimization abilities. Example prompts include: “What are the specific gaps in depth, breadth, or creativity between my work and the peer’s?” and “What might be the possible reasons for these gaps?”

### 3.2 Context and participants

This study was conducted within the course “Practical Development of E-Learning Platforms” utilizing a blended learning environment that combined face-to-face sessions in a university multimedia classroom with online activities supported by the “Chaoxing Fanya” platform. The instructional practice spanned 8 weeks and included two main tasks: “Comparative Analysis of Online Teaching Platforms” and “Platform Configuration and Secondary Development.”

Participants were 36 third-year undergraduate students majoring in Educational Technology at a normal university in China. They were divided into nine groups of four members each, and group-based learning activities were organized throughout the study. In terms of prior knowledge, all participants had completed foundational courses providing them with the basic disciplinary knowledge required for the study. Regarding cognitive characteristics, pre-course surveys and instructor observations indicated that most students demonstrated emerging autonomy in analysis and judgment. From the perspective of prior thinking-skill exposure, students had engaged with tasks such as problem analysis and solution design in earlier coursework, which offered a relevant foundation for conducting the self- and peer-assessment activities in this research.

### 3.3 Process and materials

**Figure 2:**
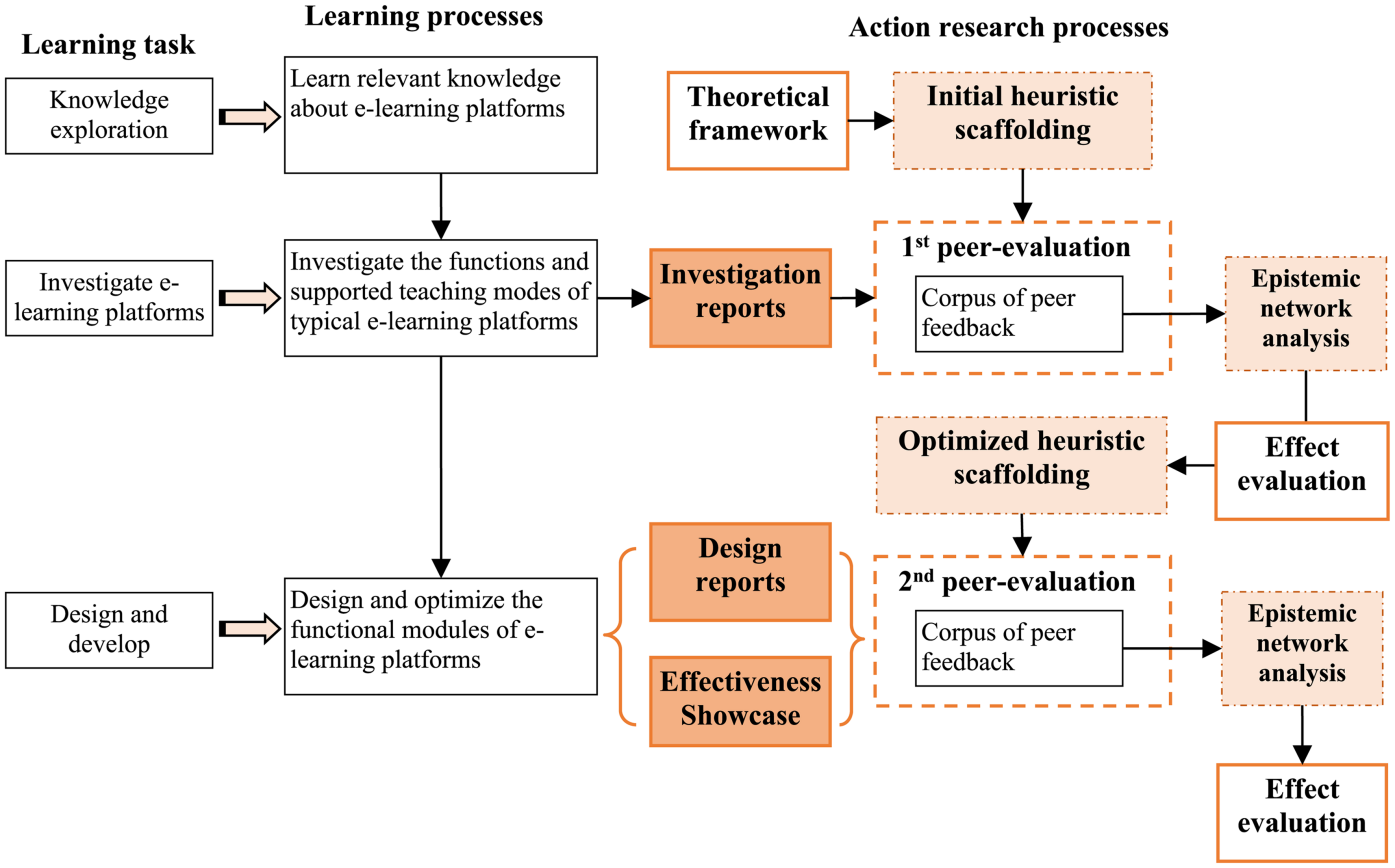
Action research processes and materials.

This study adopted an action research approach, aiming to explore, through iterative cycles of “planning-acting-observating-reflecting” the use of heuristic scaffolding to enhance students’ critical thinking skills within peer assessment. The overall process is illustrated in Figure 2.

Learners were required to complete the following tasks in sequence: first, acquire foundational knowledge regarding online teaching platforms; second, conduct an in-depth investigation and analysis of major functional modules and corresponding teaching models (e.g., self-directed learning, collaborative learning, blended learning) of typical online learning platforms (such as iCourse, Khan Academy, and Moodle) using EduTools, and identify potential shortcomings; third, attempt to redesign and redevelop modules addressing these identified deficiencies. The latter two tasks resulted in the production of a research report on online teaching platforms, a system design report, and a system demonstration video.

**Figure 3:**
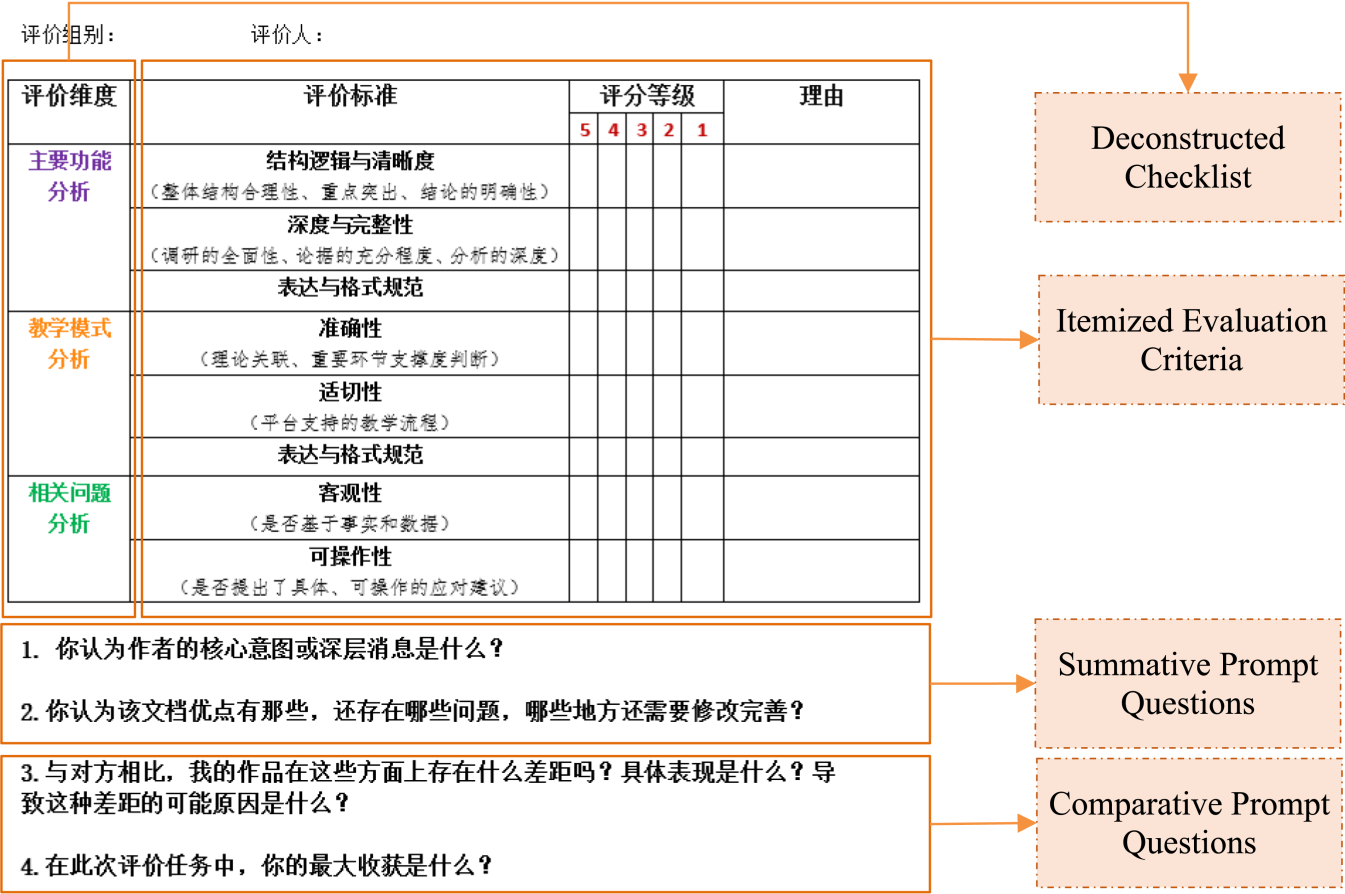
Initial heuristic scaffolding.

**Figure 4:**
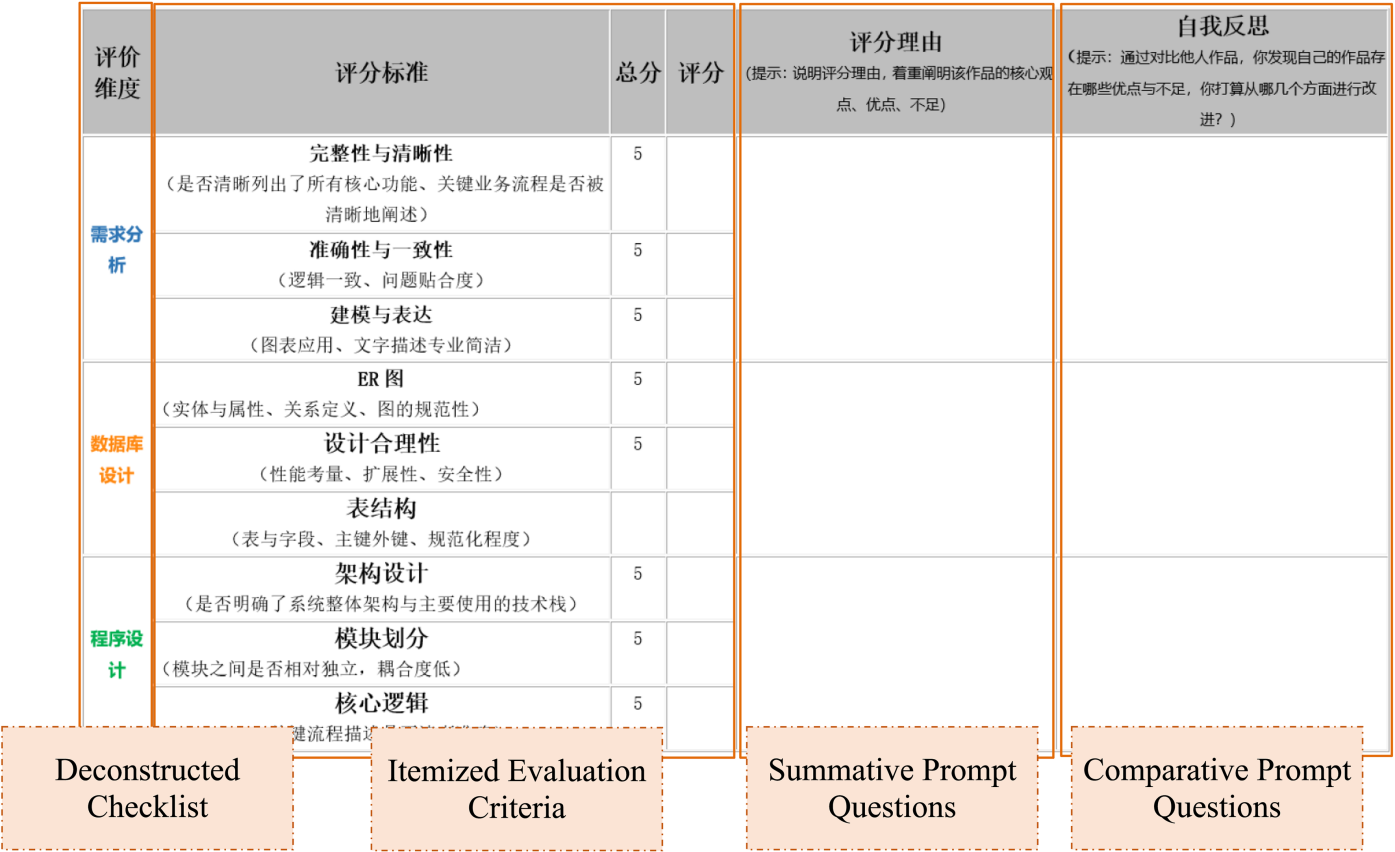
Optimized heuristic scaffolding.

Grounded in a peer assessment framework that integrates critical thinking components, an initial heuristic scaffolding system was developed (see Figure 3). Two rounds of inter-group peer review were conducted: the first focused on investigation reports, and the second on design reports and demonstration videos. During each round, peer assessment discourse was collected to build a corpus of feedback. epistemic network analysis (ENA) was used to quantitatively evaluate the scaffolding’s effectiveness. Based on the results of the first round, the scaffolding was refined and redeployed in the second round (see Figure 4).

### 3.4 Data analysis

The coding process was guided by the critical thinking analysis and coding frameworks established by Wu et al. (2015) and Leng and Guo (2018), which informed the development of a coding scheme comprising five dimensions: Analysis (A), Evaluation (E), Reasoning (R), Interpretation (I), and Self-regulation (S). Analysis refers to the systematic deconstruction of information, viewpoints, problems, or arguments to identify their underlying structures, components, and interrelationships. Evaluation involves making a deliberate judgment about the quality and validity of information, arguments, or solutions based on clear criteria. Interpretation entails articulating one’s understanding of information in a clear, accurate, and structured manner. Reasoning means drawing reasonable conclusions or predictions from available information through logical reasoning. Self-regulation refers to the metacognitive monitoring and adjustment of one’s own thinking processes. Using the sentence as the unit of analysis, two researchers independently coded the learning evaluation reports according to the coding framework (see Table 1). Inter-coder reliability, as measured by Cohen’s Kappa, was 0.763, indicating good agreement. Any coding discrepancies were resolved through joint discussion and consensus between the two coders.

**Table 1:** Critical thinking coding framework.

Components	Sub dimension	Meaning
A: Analysis	A1: Deconstruct components	Breaking down complex information into logical categories or components
	A2: Relate concepts	Identifying relationships or patterns among information
	A3: Reference evidence	Using specific examples or data to support arguments
E: Evaluation	E1: Apply standards	Systematically evaluating using appropriate criteria
	E2: Assess credibility	Judging the reliability and limitations of information sources
	E3: Check logic	Examining the logical rigor of an argument
	E4: Judge value	Making value-based judgments
I: Interpretation	I1: Interpret meaning	Uncovering deeper meanings or theoretical connections
	I2: Synthesize perspectives	Integrating different viewpoints to interpret issues
R: Reasoning	R1: Infer conclusions	Drawing conclusions based on available evidence
	R2: Recommend solutions	Proposing actionable suggestions for improvement
S: Self-regulation	S1: Reflect critically	Reflecting on strengths and weaknesses in one’s thinking process
	S2: Iterate improvements	Revising thinking and improving work based on new evidence

## 4 Results

### 4.1 Cycle 1: implementation and initial evaluation

**Figure 5:**
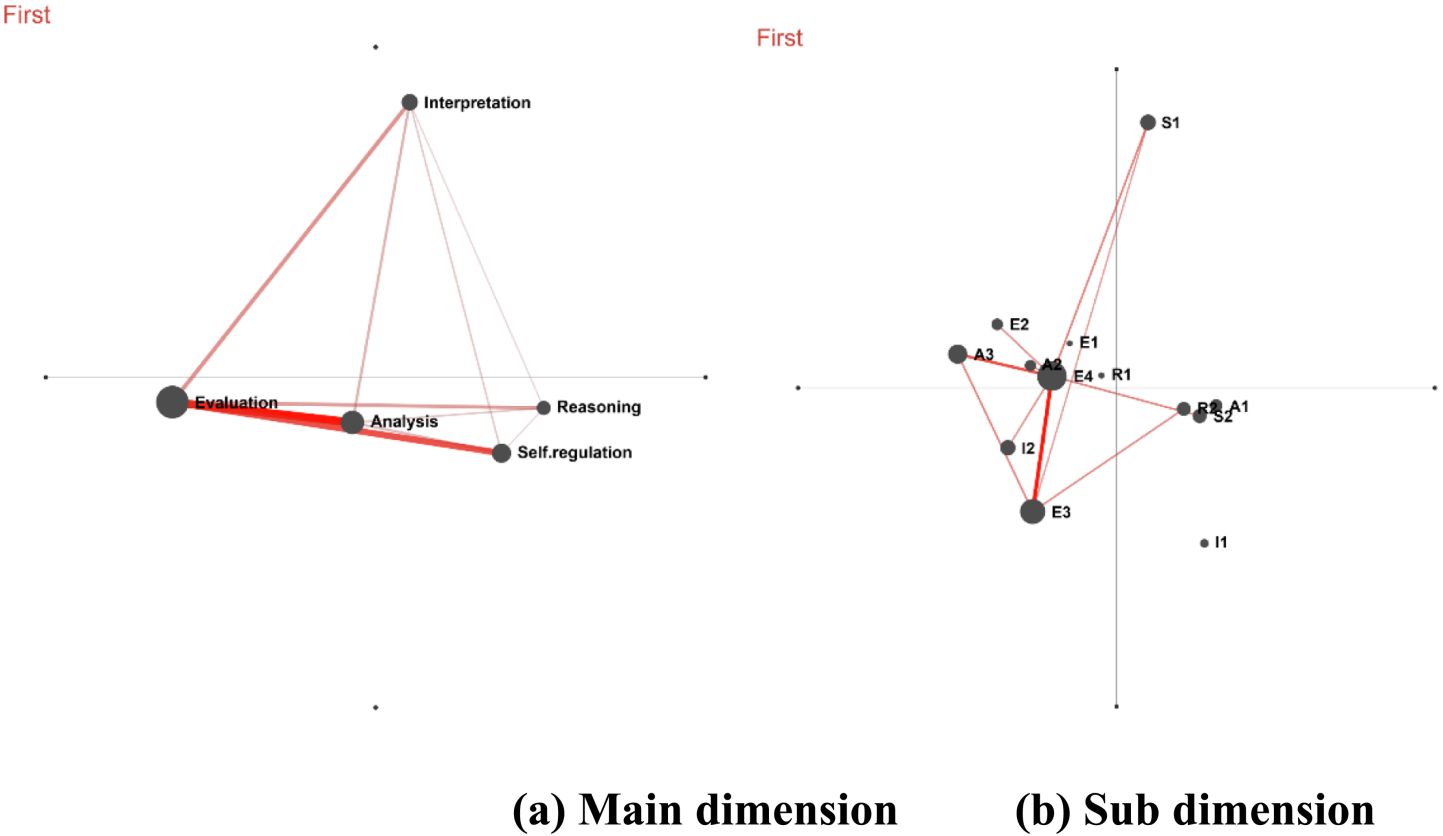
Cognitive network following the initial intervention (Cycle 1). This diagram is based on peer assessment texts from students and employs epistemic network analysis (ENA) to examine co-occurrence patterns among critical thinking elements. Nodes represent the five core elements of critical thinking, with node size corresponding to their weighted degree centrality. Edges indicate the co-occurrence of two elements within the same speech turn, and edge thickness reflects the frequency of co-occurrence.

Figure 5A illustrates the co-occurrence cognitive network of the five critical thinking elements. (1) The formation of a relatively balanced network among the five elements indicates that all core skills—Interpretation, Evaluation, Analysis, Reasoning, and Self-regulation—were activated during the peer assessment process. (2) The interconnections among these elements suggest that learners did not perform tasks in isolation but were able to integrate these skills organically. For instance, their Evaluation was grounded in Analysis and Reasoning, while their Interpretation was accompanied by Self-regulation to ensure accuracy of understanding. (3) Strong co-occurrence was observed between Analysis and Evaluation (A–E) and between Evaluation and Self-regulation (E–R), while co-occurrence among other elements was present but weaker. These results indicate that, during the first round of activities, students’ thinking processes primarily progressed from analysis to evaluation, followed by reflection on their own work, leading to strategies for further refinement and optimization.

Figure 5B displays the cognitive network of second-level indicators of critical thinking elements, revealing an uneven distribution of specific behaviors (nodes) or connections concentrated around certain nodes (e.g., a high density of Evaluation nodes, whereas Analysis or Reasoning nodes were fewer or weakly connected). This suggests that the scaffolding may have overemphasized Evaluation while underemphasizing in-depth Analysis and rigorous Reasoning. Learners’ feedback tended to focus on direct judgments (e.g., “this is not good”) without in-depth analysis or justification of “why it is not good.” The relatively low number of interpretation and reasoning nodes, along with their weak connections—often linked only to simple evaluative behaviors (e.g., connected only to E1) rather than to more complex analytical behaviors—indicates insufficient depth in Interpretation and Reasoning.

Furthermore, while the first-level indicator “Self-regulation” showed connections to other nodes, the second-level indicator network lacked specific sub-nodes representing Self-regulation. This implies that although the scaffolding stimulated macro-level metacognitive awareness, it failed to provide sufficient actionable guidance to prompt learners to engage in profound and concrete self-reflection and self-regulation.

### 4.2 Reflection and revision

The results of the first-round ENA indicated that the initially constructed heuristic scaffolding demonstrated certain effectiveness in activating the overall framework of critical thinking. However, significant deficiencies remained in promoting the development of deeper and more integrated student thinking. These shortcomings can be summarized into three main issues: first, evaluation behaviors exhibited a high density, but the depth of reasoning and interpretation behaviors—core components of critical thinking—was insufficient. Students often remained at the level of superficial judgment, failing to fully engage in evidence-based logical deduction or in-depth interpretation. Second, self-regulation behaviors lacked operational support; the related guidance remained at a conceptual level and failed to form a clear behavioral pathway, making it difficult for students to translate reflection into practical cognitive optimization actions. Third, the internal connections among the various thinking elements had not yet formed a closed loop. Specifically, there was a disconnection in the critical link from interpretation/inference to self-reflection, resulting in a fragmented thinking process that hindered the systemic operation of critical thinking.

In response to the aforementioned issues, this study systematically revised the heuristic scaffolding with the aim of effectively promoting deeper and more integrated student thinking in the second round of action research. The specific revision strategies were as follows:

(1) Addition and Optimization of Heuristic Scaffoldings Targeting Interpretation and Reasoning.

To address the lack of depth in interpretation and reasoning behaviors, the revision process moved away from traditional simplistic comprehension questions. Instead, a series of higher-order questioning scaffolds were added, designed to promote comprehensive analysis, causal attribution, and trend prediction. Through a mandatory guided questioning design, students were pushed toward deeper cognitive processing. The new questions focused on three key dimensions: constructing logical chains of causal reasoning, integrating and contrasting multiple perspectives, and evidencing and interpreting core positions. This ensured that students’ thinking activities shifted from surface-level cognition to deep logical analysis. Simultaneously, the sequence of questions was systematically optimized, strictly following the critical thinking logical chain of Interpretation → Analysis → Reasoning → Evaluation. This organization ensured that the reasoning and interpretation phases were built upon a sufficient analytical foundation, avoiding jumps and fragmentation in the thinking process.

(2) Strengthening the Internal Connections Among Thinking Elements, with a Focus on Constructing a Foundational Pathway for Self-Reflection.

Aiming at the problems of loose connections between thinking elements and the failure to ground self-regulation behaviors in practice, the core of the revision strategy was to explicitly construct a cognitive bridge from evaluating others to reflecting on oneself. Through scaffold design, students were guided to directly translate their analysis, reasoning, and interpretation of others’ work into concrete action plans for optimizing their own work, thereby achieving deep integration of thinking elements. This was concretely realized by abandoning the ambiguity of traditional open-ended questions and adopting a structured, mandatory comparative table. This table linked core dimensions such as the key points for evaluating others’ work, a comparative analysis of the differences between one’s own work and others’, and specific self-improvement measures based on the comparison. This design not only helped students accurately understand others’ work (knowing what), but also prompted them to deeply grasp the rationale behind evaluations (knowing why), ultimately enabling the transformation of evaluative conclusions into practical actions for self-regulation (reflecting on oneself). This fostered the iterative optimization of both thinking and outputs, forming a complete, in-depth, and tightly connected critical thinking closed loop.

### 4.3 Cycle 2: revised implementation and outcomes

**Figure 6:**
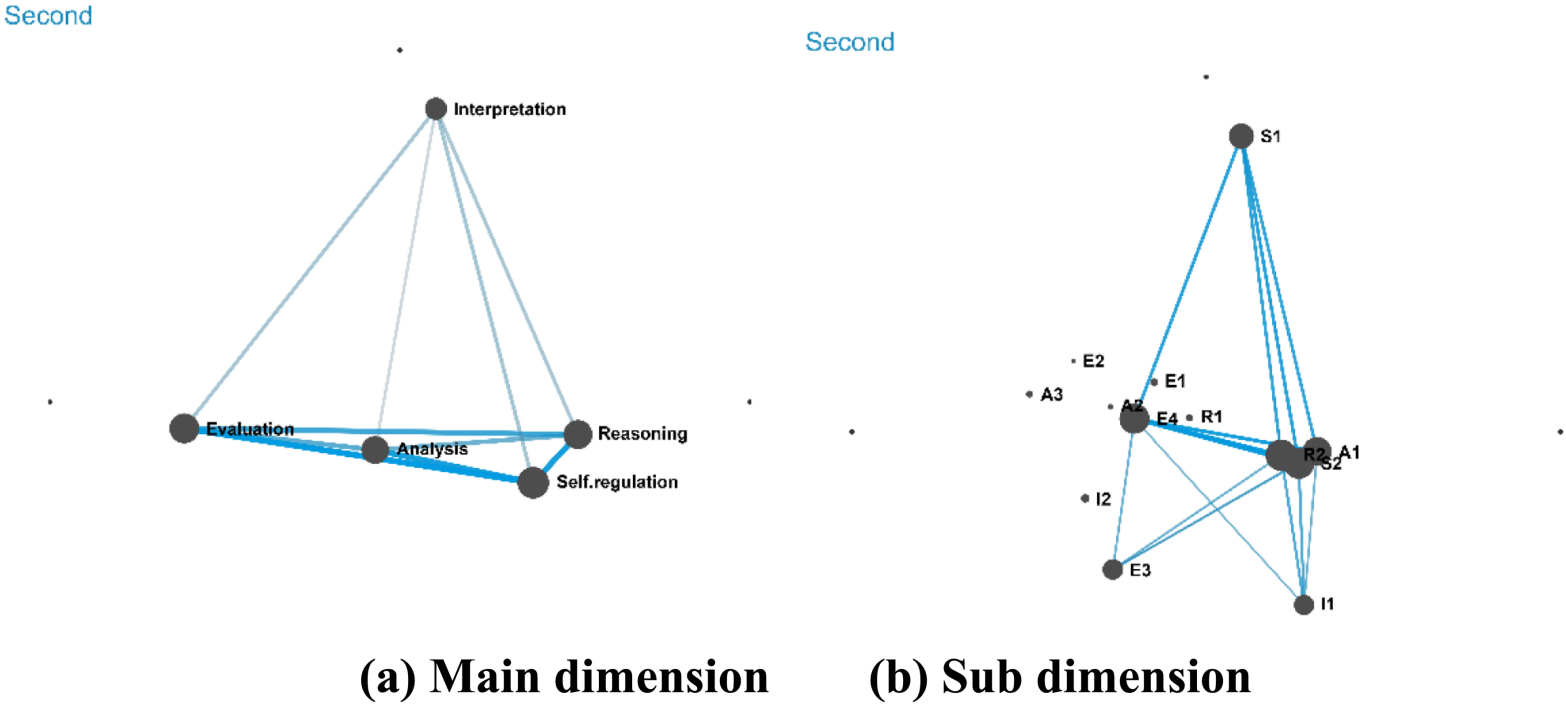
Cognitive network following the second intervention (Cycle 2).

Following the revisions made to the scaffolding in the second round, the ENA network (see Figure 6) revealed tighter and more structured connections among the elements of critical thinking, indicating that the refined heuristic scaffolding effectively promoted the integration and deepening of students’ critical thinking during peer assessment. Specific improvements are reflected in the following aspects:

(1) Significant deepening of reasoning and interpretation behaviors: second-level indicators such as R1 (Infer Conclusions) and R2 (Recommend Solutions) showed strong associations with the first-level Reasoning node. This suggests that the added high-order prompts (e.g., constructing causal chains, integrating multiple perspectives) effectively guided students to move beyond surface-level judgments toward deeper logical deduction. In evaluating peers’ work, students placed greater emphasis on evidence-based inference and solution-oriented thinking.
(2) Evaluation behaviors with stronger logical support: Second-level behaviors including E2 (Assess Credibility), E3 (Check Logic), and E4 (Judge Value) were closely linked to the first-level Evaluation node. This demonstrates that by restructuring question sequences to connect interpretation, analysis, reasoning, and evaluation organically, students were able to systematically use analytical outcomes as a basis for evaluation, thereby reducing subjective judgment and enhancing the rationality and persuasiveness of their assessments.
(3) Operationalization of self-regulation: S1 (Reflect Critically) and S2 (Iterate Improvements) formed a feedback loop with behaviors such as Evaluation and Reasoning in the ENA network. This indicates that through the introduction of the Comparative Metacognitive Guidance Table, students could translate their evaluations of others (e.g., analyzing argument logic, judging evidence validity) into reflective insights and concrete steps for improving their own work. This transition from “assessing others” to “revising one’s own work” enhanced actionable self-regulation.
(4) Enhanced systemic thinking and reduced fragmentation: connections among first-level nodes became denser and more coherent, particularly among Reasoning, Interpretation, and Self-regulation. This shows that the revised scaffolding—through mandatory structured guidance such as comparative tables and integrated question design—helped students establish a clear thinking process, facilitating the development of critical thinking toward greater depth, integration, and cyclical refinement.

**Figure 7:**
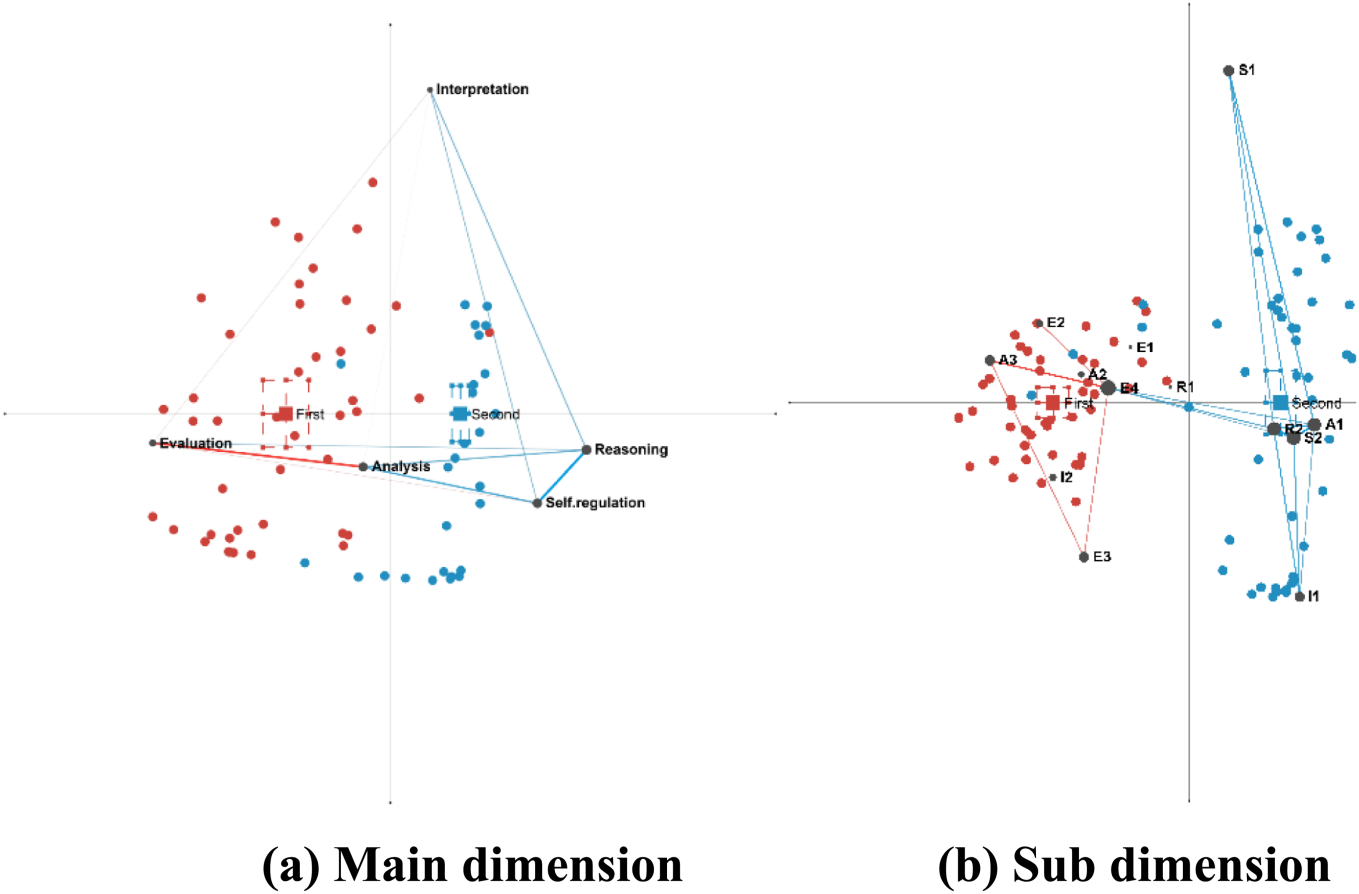
Cognitive network difference between two rounds of activities. This figure visualizes the dynamics of cognitive structure by subtracting the first-round ENA network from the second-round network. Blue elements represent the second round, while red elements denote the first round. Solid blue lines indicate strengthened cognitive connections in the second round, and solid red lines signify weakened connections. Node size reflects the magnitude of change in element centrality, and edge thickness corresponds to the absolute change in co-occurrence frequency. The red dashed rectangle marks the centroid of the first round, and the blue dashed rectangle indicates the centroid of the second round.

Using ENA, a cognitive network difference map (First–Second) was plotted (see Figure 7) to analyze changes in students’ critical thinking characteristics between the two rounds. The figure shows that The confidence intervals for the first and second rounds are independent and non-overlapping, with the interval range narrowing in the second round. The distribution surrounding the centroid of the cognitive network has become increasingly concentrated, and the centroid position has undergone a shift. These changes indicate a systematic transition in students’ cognitive structure from an analysis-evaluation orientation toward an evaluation-inference-self-regulation orientation, accompanied by increased convergence in cognitive patterns.

The results of the network subtraction analysis indicate that during the initial round, alterations in the analysis–evaluation pathway manifested to a greater extent than in the subsequent round. In contrast, the second round exhibited substantially stronger changes in the evaluation–inference, analysis–self-regulation, interpretation–inference, and inference–self-regulation pathways. Within the critical thinking process, the four elements—analysis, evaluation, inference, and self-regulation—exhibit a hierarchical relationship, progressing from lower-order to higher-order thinking. These findings demonstrate that as the activity progressed, students’ critical thinking shifted from lower-order processes (analysis–evaluation) toward higher-order processes (evaluation–inference–self-regulation).

## 5 Discussion

Critical thinking is characterized by a reflective and questioning attitude toward information, as well as the ability to analyze, judge, and make decisions. This study designed peer assessment activities based on heuristic scaffolding to guide learners beyond superficial evaluation and engage them in a deep learning process involving multiple cognitive dimensions. During this process, learners encounter perspectives that differ from their own cognitive structures while evaluating others’ work, thereby stimulating cognitive conflict. The heuristic scaffolding further guides learners to identify and reconcile such conflicts, promoting active information integration and cognitive reconstruction (Barahona et al., 2023).

Text coding analysis reveals that as the peer assessment activities progressed, the frequency of higher-order thinking codes increased consistently, reflecting a systematic enhancement in students’ cognitive levels (Leng and Guo, 2018; Zhang et al., 2022). Epistemic network analysis (ENA) results further demonstrate that students’ critical thinking structures became more systematic, showing a clear progression from lower-order to higher-order cognitive processes. Together, this evidence indicates that the “peer assessment + question scaffolding” intervention model developed in this study has a positive and verifiable effect on fostering critical thinking.

The mechanism underlying this model lies in the coordinated activation of multiple cognitive dimensions. When responding to guided questions during peer assessment, learners simultaneously engage key processes such as conflict recognition, social cognition, and metacognitive regulation. Heuristic questions help learners recognize discrepancies between their own views and those of others, thereby triggering conflict recognition. In attempting to reconcile contradictions and seek reasonable explanations, learners naturally engage in analysis and judgment, forming the foundation of critical thinking. Furthermore, as a social-cognitive activity, peer assessment encourages learners to understand diverse perspectives, compare and integrate different viewpoints, thereby expanding the breadth and depth of their thinking. The question scaffolding, through metacognitive guidance, prompts learners to continually examine their own reasoning and plan improvements to their work, achieving a transition from “evaluating others” to “optimizing oneself” and completing the closed-loop construction of critical thinking.

In summary, cognitive conflict serves as an internal driver for the development of critical thinking, while systematic reflection promotes the resolution of conflict and the restructuring of thinking. Therefore, effective heuristic scaffolding should integrate the dual functions of stimulating cognitive conflict and guiding systematic reflection, thereby fostering the systematic development of students’ critical thinking in authentic, collaborative learning contexts.

## 6 Conclusion

This study constructed and validated a heuristic scaffolding framework for peer assessment through action research design and epistemic network analysis. The findings indicate that the framework effectively activates the five core components of learners’ critical thinking—analysis, evaluation, interpretation, reasoning and self-regulation—and promotes the formation of an interconnected cognitive network. This transforms peer assessment from a simple evaluative task into a cognitive activity that integrates higher-order thinking skills.

An in-depth analysis of the first round of peer assessment revealed that although students’ macro-level critical thinking components were activated, a tendency toward “emphasizing evaluation over analysis and reasoning” persisted. Furthermore, self-regulatory behaviors were not fully translated into observable reflective actions. Building on these insights, the optimized scaffolding used in the second round—through strengthened analytical deconstruction checklists, comparative reflection prompts, and inferential questioning—significantly enhanced the connections among the thinking components. Specifically, it reinforced the “analysis–reasoning–evaluation” cognitive chain and facilitated students’ ability to translate external feedback into concrete plans for improving their own work, marking progression toward more advanced levels of critical thinking.

In terms of theoretical contribution, this study unveils the micro-level mechanisms through which peer assessment promotes the development of critical thinking—namely, by using structured scaffolding to trigger cognitive conflict and guide systematic reflection, thereby fostering the integration and progression of thinking components. This provides detailed empirical support for cultivating critical thinking from a social constructivist perspective. Furthermore, the scaffolding framework developed and validated in this study offers a replicable implementation model for operationalizing classical critical thinking theories, such as Facione’s model, in authentic educational contexts.

At the practical level, the findings suggest that implementing peer assessment in the context of Chinese higher education requires careful consideration of its cultural and educational characteristics. Traditional Chinese classroom culture emphasizes respect for authority and collective harmony, which may lead students to adopt a mild, suggestive feedback style during peer assessment, avoiding direct questioning or confrontation. This could potentially weaken the intensity and depth of cognitive conflict. Therefore, scaffolding design must strike a balance between stimulating intellectual clash and maintaining a collaborative atmosphere—for instance, by using tools such as “perspective comparison tables” or “evidence-based justification guides” to encourage reasoned critique within a respectful framework. Additionally, as Chinese students have often been educated in teacher-centered instructional environments, they may initially exhibit lower trust in the authority and value of peer feedback. Thus, it is essential to gradually build students’ cognitive recognition and willingness to participate through teacher modeling, transparent evaluation criteria, and procedural incentives.

This study has several limitations. The relatively small sample size and its concentration within a single discipline limit the generalizability of the conclusions. Future research could validate the applicability of this scaffolding across different disciplines, cultural contexts, and educational stages. Moreover, integrating multimodal data—such as interviews, reflective journals, and behavioral tracking—could provide a more comprehensive understanding of the internal processes and affective factors involved in the development of critical thinking. Furthermore, while the current scaffolding remains static in design, future studies could explore the integration of artificial intelligence technologies to offer dynamic, personalized thinking guidance based on learners’ real-time performance, thereby enabling more adaptive pathways for fostering critical thinking.

## Data Availability

The original contributions presented in the study are included in the article, further inquiries can be directed to the corresponding author.
